# Burden of HPV-Related Hospitalization in Germany from 2000 to 2021

**DOI:** 10.3390/v15091857

**Published:** 2023-08-31

**Authors:** Georgios Tampakoudis, Olympia E. Anastasiou

**Affiliations:** 1 Maternal-Fetal Medicine and Obstetrics Saint Luke’s Hospital, 55236 Thessaloniki, Greece; georgetampakoudis@gmail.com; 2 First Department of Obstetrics and Gynaecology Papageorgiou General Hospital, Aristotle University of Thessaloniki, 54124 Thessaloniki, Greece; 3 Institute for Virology, Essen University Hospital, University of Duisburg-Essen, 45147 Essen, Germany

**Keywords:** HPV, cervical cancer, hospitalization rate, head and neck cancers, anal cancer, penile cancer, oropharyngeal cancer, vulva cancer, vaginal cancer, inpatient mortality

## Abstract

HPV has been linked to the development of precancerous and cancerous lesions. The aim of this study was to evaluate the burden of HPV-related hospitalization in Germany from 2000 to 2021 and the potential impact of the COVID-19 pandemic on it. Methods: We performed a retrospective query using data from the German Statistical Office from 2000 to 2021, including hospital admission, inpatient mortality and hospital stay length data on cervical cancer/dysplasia, female genitourinary tract, anal, penile, head and neck cancers. Results: The HPV-attributable hospitalization rate per 100,000 inhabitants in Germany has decreased over time, from 89 cases in 2000 to 60 in 2021, with an average annual percent change (AAPC) of −1.93 (CI −2.08–−1.79, *p* < 0.05). The same trend was observed for the average hospital stay, which declined from 9 to 7 days, with an AAPC of −1.33 (CI −1.52–−1.21, *p* < 0.05). An undulating but overall slightly declining pattern was observed for the inpatient mortality (AAPC −0.92, CI −1.21–−0.64, *p* < 0.05). We observed a reduction in the hospitalization rates for invasive and non-invasive cervical cancer, which was observed in almost all age groups and in all German federal states. Conclusion: Our study provides a comprehensive analysis of the trends in HPV-related hospitalizations over the past two decades. The decline in hospitalization rates for cervical cancer and dysplasia suggests the potential efficacy of the HPV vaccination and screening programs.

## 1. Introduction

Human papillomaviruses (HPV) are a family of viruses that can cause benign and malignant cutaneous and mucosal lesions and have been linked to various diseases, including anogenital dysplasia, cancer and upper aerodigestive tract neoplasms. Man is the only known natural reservoir. They are transmitted through skin or mucosa contact through micro-injuries, in most cases through person-to-person contact. The virions infect the epithelial cells of the basal cell layer. Sexual contact is the main mode of transmission. Vaginal or anal sexual intercourse is the transmission route for infections in the anogenital area, while transmission into the oral cavity or the oropharynx is possible via orogenital sexual practices. Condom use does not entirely negate the risk of transmission. Rarely, HPV can also be transmitted through a smear infection or from mother to newborn peripartum. Most HPV infections produce no symptoms [1,2].

More than 200 HPV genotypes have been identified. However, the most clinically important classification within the HPV family is that of high vs. low risk, according to the oncogenic potential of each type. Twelve high-risk subtypes are currently classified as definitely carcinogenic: 16, 18, 31, 33, 35, 39, 45, 51, 52, 56, 58 and 59. The most important one, from an epidemiological point-of-view, is the high-risk HPV type 16, which can be detected in the majority of HPV-related carcinomas [1,2]. Cofactors for the development of HPV-related malignancy include smoking, use of hormonal contraceptives, multiple pregnancies and immunosuppression [1].

HPV infection has been linked to considerable morbidity and mortality due to its carcinogenic potential and was estimated to have caused almost half a million cases and 250,000 deaths from cervical cancer in 2002 [3]. Of 14 million new cancer cases in 2012, 640,000 (4.5%) cases were attributable to HPV [4], 8.6% in women and 0.8% in men [5]. Among women, cervical cancer ranked fourth in global cancer statistics, for both incidence and mortality [6]. The incubation time between persistent infection with high-risk HPV types and the development of high-grade cervical dysplasia is estimated at 3–6 years, while developing an invasive cervical carcinoma takes an additional 10 to more than 30 years. There are no reliable data on the time frame from persistent infection with high-risk HPV types to tumor development in men [2]. Persistence is a necessary but not a sufficient condition for carcinogenesis, since there are non-carcinogenic HPV types, such as HPV 61, that can establish a persistent infection, but do not cause cancer [1]. Overexpression of HPV oncoproteins is crucial for carcinogenesis, as this leads to the inactivation of tumor-suppressor proteins and disruption of growth-regulatory intracellular signaling pathways. This is often but not always the result of the integration of the viral genome into the genome of the host cell. Persistent infections with high-risk HPV increase the risk of integration [2].

Data from 2010 indicate that 38% of young German women are infected with HPV of any type, most (90%) of which are at a high-risk for cancer type [7]. According to a 2020 report, the annual number of cervical cancer cases and deaths in Germany is 4666 and 2075, respectively [8]. Two prophylactic HPV vaccines have been available since 2006 [2] and have been incorporated in the German universal immunization plan since 2007, initially for girls aged 12–17 [9]. The recommendation was lowered to 9–14 years in 2014. Since 2018, it has been recommended for both girls and boys at the age of 9, with catch-up vaccination until the age of 17 years [2,10].

The aim of this study was to evaluate the burden of HPV-related hospitalization in Germany from 2000 to 2021 and the potential impact of the COVID-19 pandemic on it.

## 2. Patients and Methods

We performed a retrospective query using data from the German Statistical Office (Statistisches Bundesamt Deutschland) [11]. The database contains data from all hospital discharges from 2000 to 2021. The database includes information on medical data, such as primary diagnosis, length of stay and inpatient mortality, and demographical data, such as population size, sex and age groups. Primary diagnoses are codified according to the ICD-10-GM coding system to a three-character level. Data are available in an aggregated format. The query for HPV cancer-related disease in hospitalized patients included the following ICD-10-GM codes: C00 to C14 (oral and oropharyngeal cancer), C21 (anal cancer), C51 (cancer of the vulva), C52 (vaginal cancer), C53 (cervical cancer), C60 (penile cancer, D06 (carcinoma in situ of the cervix) and N87 (cervical dysplasia).

According to published literature, the following attributable fractions (AFs) were used to quantify the proportion of hospitalizations attributable to HPV for each disease group: 100% for cervix cancer, 88% for anal cancers, 77% for female genitourinary tract cancers, 50% for penile cancers and 26% for oral and pharyngeal cancers [12,13,14]. Our analysis did not include genital warts, although HPV plays a central role in their pathology, because the three-character-ICD-10-GM they belonged to was not unique to them. However, genital warts are a rare primary diagnosis for inpatients, with only 54 cases in 2019 (data from InEK DatenBrowser, assessed on 12 July 2023). The total HPV-attributable hospitalizations were thus calculated by multiplying each specific AF with the total number of hospitalizations for that condition and adding the results of all the above-mentioned conditions.

The hospitalization rate was calculated per 100,000 people based on the German population size each year. Statistical analyses were performed using SPSS 19.0. The time trends were analyzed using the Joinpoint model (Joinpoint version 5.02, May 2023) to evaluate the direction and the intensity of the (linear) trend, estimating an annual percent change (APC) and the average annual percent change (AAPC). AAPC is a summary measure of the trend over a pre-specified fixed interval, describing the average annual percent change (APC) over a period of multiple years. It is computed as a weighted average of the APCs from the Joinpoint model, with the weights equal to the length of the APC interval. The final model is based on linear segments connected at joinpoints, representing the best fit of the observed data [15,16]. The level of significance was set to *p* < 0.05. The term “stable” was used in the event that the APC was between −0.5 and 0.5. Below this interval, trends were deemed “decreasing”; above this interval, they were deemed “increasing”. Ethical review and approval were waived for this study, since it involved aggregated, publicly available data.

## 3. Results

### 3.1. The Hospitalization Rate of HPV Attributable Diseases Has Decreased over Time

**Figure 1:**
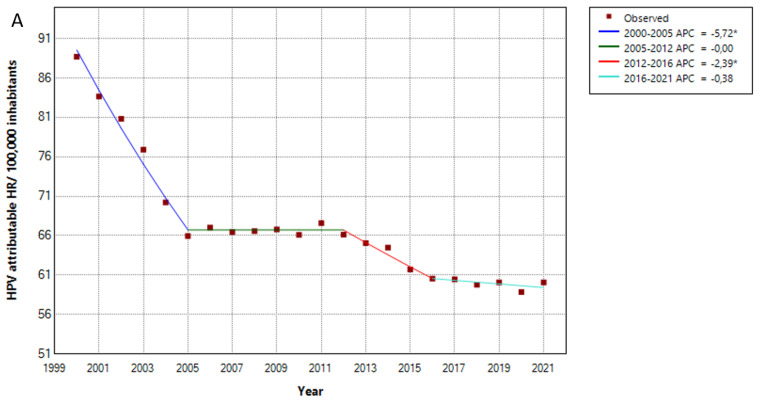

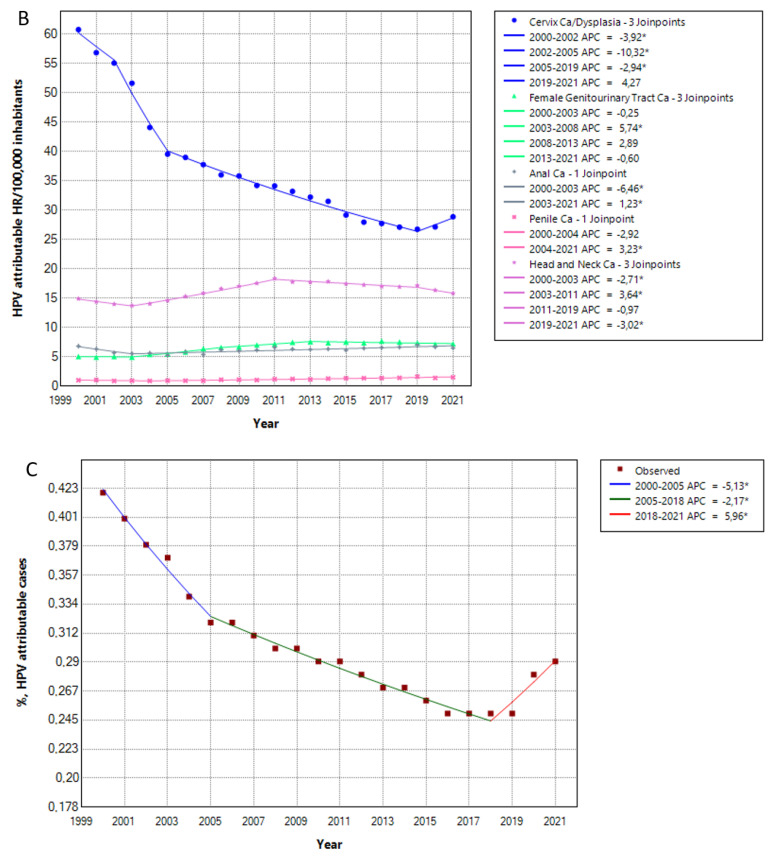
HPV-related hospitalization rate per 100,000 inhabitants from 2000 to 2021, in total (**A**), after stratification according to cause (**B**). The percentage of HPV-attributable hospitalized cases to the total number of hospitalizations (**C**). APC: annual percentage rate, Ca: cancer, HR: hospitalization rate. * indicates that the APC is significantly different from zero at the alpha = 0.05 level.

The HPV attributable hospitalization rate per 100,000 inhabitants in Germany has decreased over time, from 89 cases/100,000 inhabitants in 2000 to 60 in 2021. We observed four distinct phases: a rapid decrease from 2000 to 2005, a lengthy stabilization phase from 2005 to 2012, another decrease from 2012 to 2016 and a stable phase since 2016, including the COVID-19 pandemic period. The AAPC was −1.93 (CI −2.08–−1.79, *p* < 0.05) (Figure 1A). As seen in Figure 1B, this decrease could be mostly attributed to a large decrease in cervical cancer or dysplasia cases, while the number of HPV-attributable female genitourinary tract, anal, penile, head and neck cancer cases increased or remained stable over time. The AAPC for HPV-related cases was −3.5 (CI −3.81–−3.17, *p* < 0.05) for cervical cancer/dysplasia, 1.76 (CI 1.51–2.03, *p* < 0.05) for female genitourinary tract cancer, 0.1 (CI −0.26–0.54, *p* ≥ 0.05) for anal cancer, 2.03 (CI 1.48–2.91, *p* < 0.05) for penile cancer and 0.31 (CI 0.16–0.47, *p* < 0.05) for head and neck cancers. Additionally, the percentage of HPV-related cases to the total number of hospitalizations decreased over time from 2000 to 2018, but demonstrated a sharp rising tendency during the COVID-19 pandemic. The AAPC was −1.77 (CI −1.97–−1.58, *p* < 0.05) (Figure 1C).

### 3.2. Inpatient Death Rates for HPV Attributable Diseases Undulate over Time

**Figure 2:**
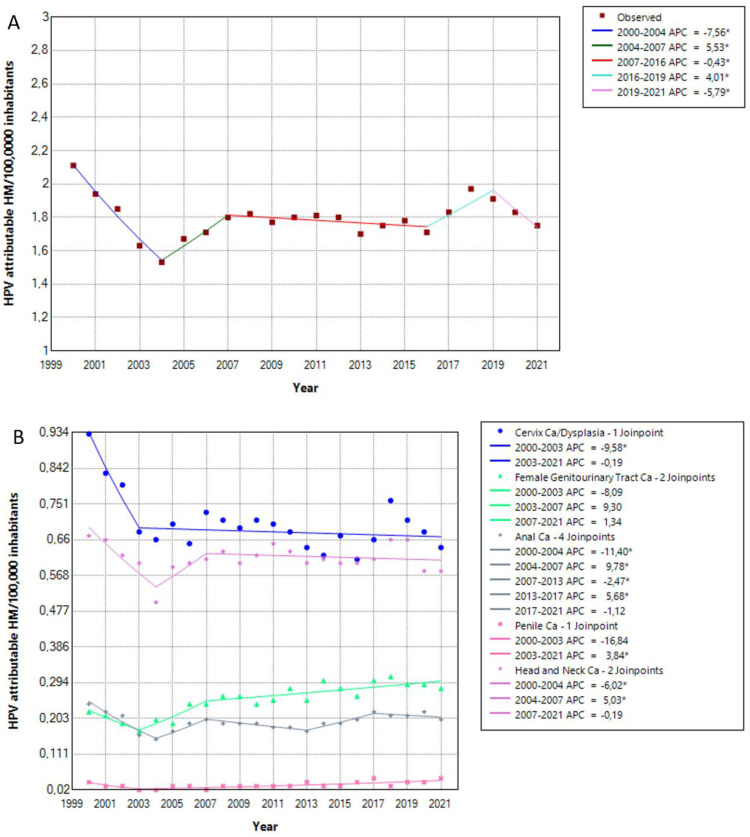

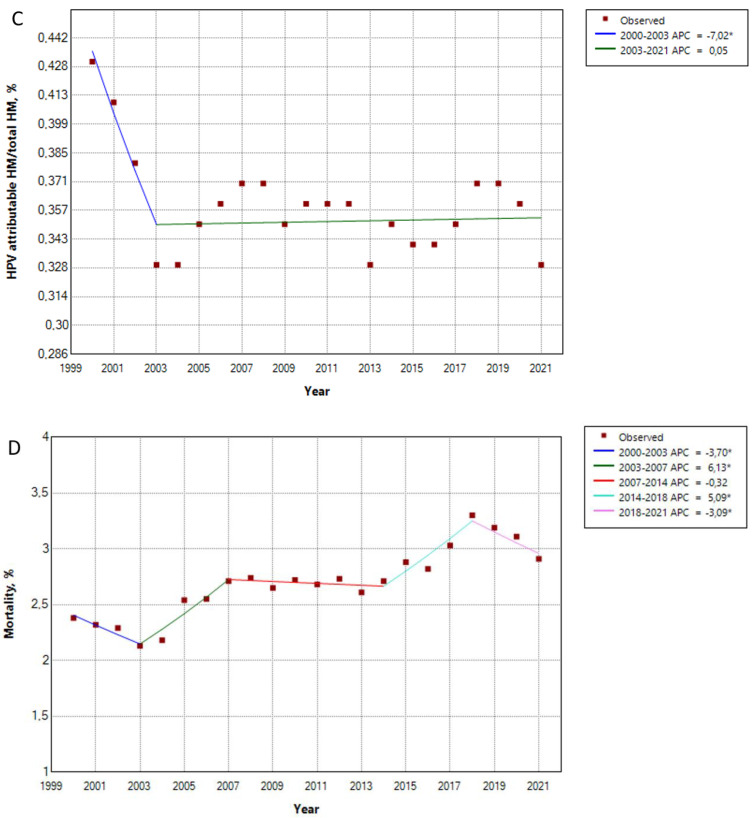
HPV-related inpatient mortality per 100,000 inhabitants from 2000 to 2021 in total (**A**), after stratification according to cause (**B**). The percentage of HPV-attributable fatal inpatient cases to the total number of fatal hospitalizations (**C**), inpatient mortality, defined as the percentage of HPV-attributable fatal inpatient cases to the total number of HPV-attributable hospitalizations (**D**). APC: annual percentage rate, Ca: cancer, HM: hospital mortality. * indicates that the APC is significantly different from zero at the alpha = 0.05 level.

The inpatient mortality decreased slightly from 2000 to 2021, with an AAPC of −0.92 (CI −1.21–−0.64, *p* < 0.05). After an initial decrease in HPV-attributable inpatient mortality from 2000–2004, the rate increased again from 2004 to 2007 and remained stable from 2007 to 2016. Another increase was seen from 2016 to 2019 with an accompanying decrease during the pandemic, from 2019 to 2021 (Figure 2A). The initial dipping from 2000 to 2003 or 2004 could be observed irrespective of disease group. Afterwards, we saw a stabilization with slight decreasing or increasing tendencies. The AAPC for HPV-related cases was −1.59 (CI −2.11–−0.63, *p* < 0.05) for cervical cancer/dysplasia, 1.39 (CI 0.59–2.64, *p* < 0.05) for female genitourinary tract cancer, −0.84 (CI −1.35–0.41, *p* < 0.05) for anal cancer, 0.59 (CI −0.9–3.22, *p* ≥ 0.05) for penile cancer and −0.61 (CI 1.1–0.18, *p* < 0.05) for head and neck cancers (Figure 2B). The same slight declining trend could be observed for the percentage of HPV-related inpatient fatal cases to the total fatal inpatient cases with an AAPC of −0.99 (CI −1.35–−0.49, *p* < 0.05) (Figure 2C), while inpatient mortality, defined as the percentage of HPV attributable fatal inpatient cases to the total number of HPV attributable hospitalizations showed a slightly increasing tendency over time with an AAPC of 0.99 (CI 0.59–1.36, *p* < 0.05) (Figure 2D).

### 3.3. The Average Hospital Stay for HPV Attributable Cases Decreased over Time

**Figure 3:**
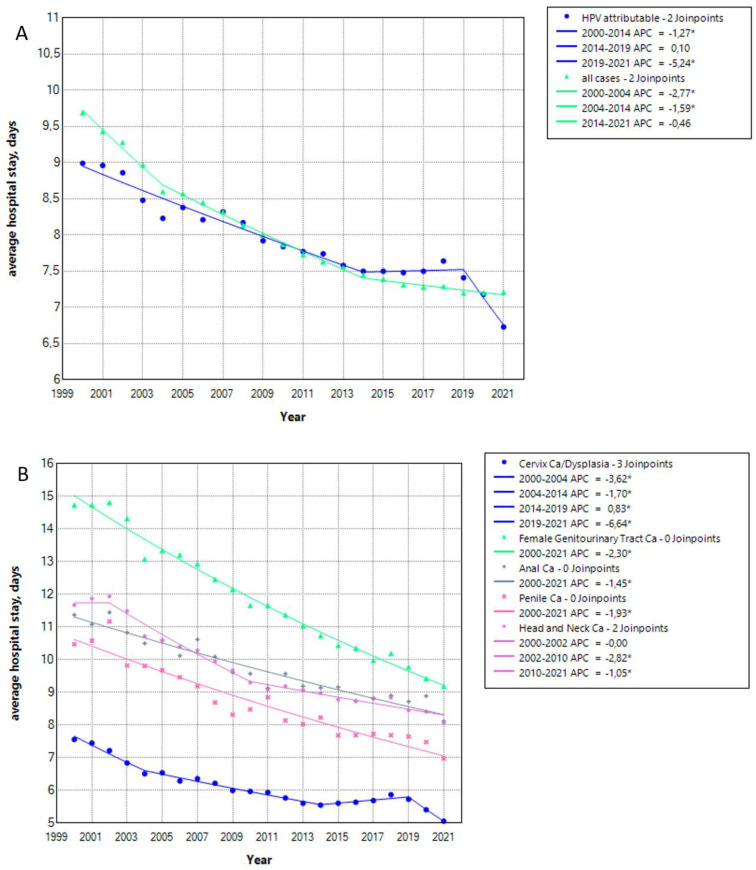

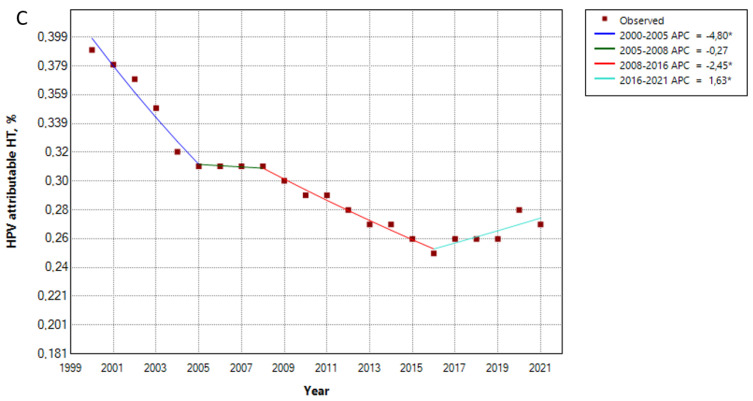
Average hospital stay of HPV-attributable hospitalized cases in total (**A**), after stratification according to cause (**B**). The percentage of hospital stay in days in HPV-attributable cases to the total hospital stay of all cases (**C**). APC: annual percentage rate, HT: hospitalization time, Ca: cancer. * indicates that the APC is significantly different from zero at the alpha = 0.05 level.

The average hospital stay for HPV-attributable cases was similar to that for all hospitalized cases (all causes) and decreased over time with an AAPC of −1.33 (CI −1.52–−1.21, *p* < 0.05). The time decreased from 9 to 7 days in 2000 and 2021, respectively. For reference, the AAPC for all hospitalized cases was −1.44 (CI −1.5–−1.37, *p* < 0.05). The decrease was steady from 2000 to 2014, and entered a stable phase from 2014 to 2019, followed by a steep decrease in the pandemic period from 2019 to 2021, as seen in Figure 3A. This trend could be observed in all evaluated pathologies. The AAPC was −1.96 (CI −2.19–−1.8, *p* < 0.05) for cervical cancer/dysplasia, −2.3 (CI −2.44–2.16, *p* < 0.05) for female genitourinary tract cancer, −1.45 (CI −1.67–−1.25, *p* < 0.05) for anal cancer, −1.93 (CI −2.23–−1.64, *p* < 0.05) for penile cancer and −1.63 (CI −1.85–−1.45, *p* < 0.05) for head and neck cancers (Figure 3B). Interestingly, the average hospital stay for female genitourinary tract cancer was the highest, twice as high as that for cervical cancer/dysplasia, while the average stay for other causes was reported somewhere in between (Figure 3B). The percentage of hospital stays in days in HPV-attributable cases to the total hospital stay of all cases decreased from 2000 to 2016 to varying degrees, and rose slightly from 2016 to 2021. The AAPC was −1.76 (−1.99–−1.53, *p* < 0.05) (Figure 3C).

### 3.4. Cervical Cancer Hospitalization Rates Have Decreased over Time but to a Different Degree in Different Federal States

**Figure 4:**
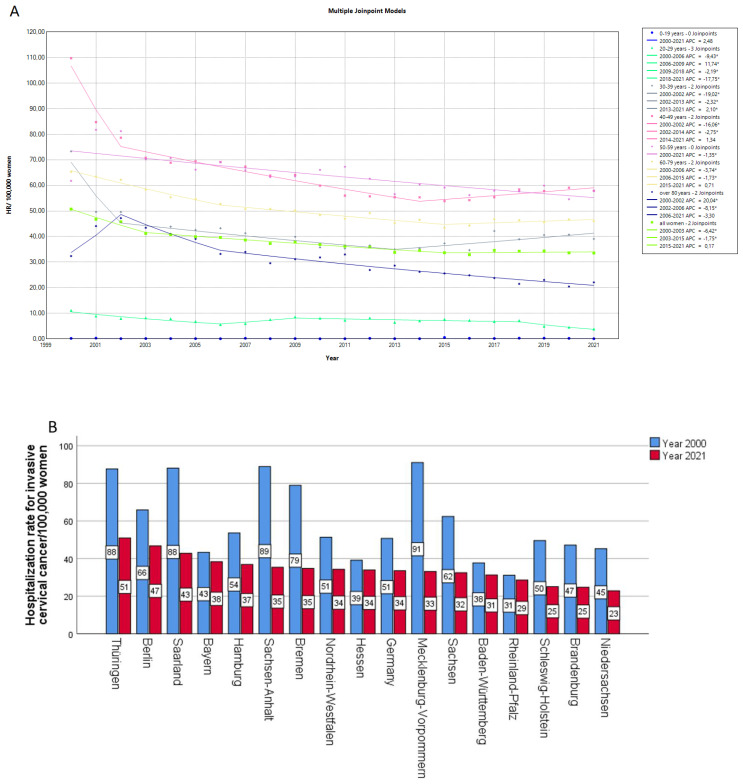

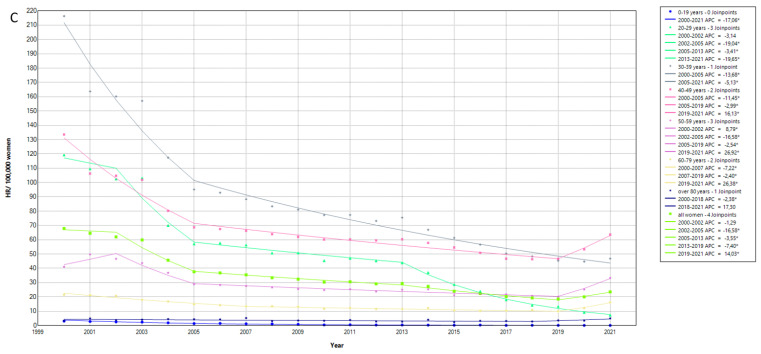
Hospitalization rate of invasive cervical cancer per 100,000 women stratified according to their age group over time (**A**), according to the federal state for the years 2000 and 2021 (**B**). Hospitalization rate of non-invasive cervical cancer and cervical dysplasia per 100,000 women stratified according to their age group over time (**C**). APC: annual percentage rate, HR: hospitalization rate. * indicates that the APC is significantly different from zero at the alpha = 0.05 level.

The hospitalization rates for invasive cervical cancer have decreased over time, with the age group of 40–69 years presenting consistently with the highest rates. In the 20–29 age group, which contains individuals who have been vaccinated against HPV, we observed a steep decreasing trend for the years 2018 to 2021, with an APC of −17.8 (Figure 4A). Furthermore, we observed that the decrease in hospitalization rate was present in all German federal states, but was more pronounced in Mecklenburg-Vorpommern, Sachsen-Anhalt and Bremen. The lowest hospitalization rate in 2021 could be observed in Niedersachsen and the highest in Thüringen (Figure 4B). The hospitalization rates for non-invasive cervical cancer or dysplasia have also decreased over time, with the 30–39 age group presenting with the highest rates. In the 20–29 years cohort, we observed a marked decrease from 2013 to 2021, with an APC of −19.7 (Figure 4C). The AAPC for the hospitalization rates for invasive cervical cancer and non-invasive cervical cancer or dysplasia are reported in Table 1.

## 4. Discussion

The hospitalization rate of HPV-related diseases in Germany has declined over the studied period, from 89 cases per 100,000 inhabitants in 2000 to 60 cases per 100,000 inhabitants in 2021. This decline in hospitalization rates could be mainly attributed to a significant reduction in cervical cancer and dysplasia cases. This decreasing trend is consistent with previous studies from other European countries [17,18,19,20]. The hospitalization rates for female genitourinary and penile cancer increased, while the rates for anal and head and neck cancers remained overall stable. The trends for the hospitalization rates of the above-mentioned diseases are less consistent in the literature, with studies reporting an increase, a decrease or a stable status [10,14,17,18,19,21,22,23].

The HPV-related inpatient mortality decreased slightly from 2000 to 2021. After an initial decrease from 2000–2004, the rate had an undulating course over time. While an overall declining trend could be observed for cervical cancer/dysplasia, anal, head and neck cancer, the opposite was true for female genitourinary and penile cancer. Additionally, the percentage of HPV-attributable fatal inpatient cases to the total number of HPV-attributable hospitalizations showed an increasing tendency over time. Data on inpatient mortality are limited in the literature, but the overall mortality rates (not inpatient mortality) for cervical cancer have been consistently decreasing in Western Europe [24]. In contrast to the slight decrease in anal cancer inpatient mortality rates seen in our cohort, the reported overall mortality rates have increased for anal cancer in the USA [25] while the mortality rate for oropharyngeal cancer has increased in men but decreased in women in the USA [26]. The incidence-based mortality rate for penile cancer showed an increasing trend in the USA [27], consistent with our results, while the mortality rate increased for vulva cancer and decreased for vaginal cancer in Poland [28]. It must be stressed that our data include only inpatient mortality, whereas overall mortality as reported in previously cited studies includes all cases, irrespective of inpatient or outpatient status. Thus, a direct comparison of mortality rates would be problematic. Apart from the decreasing tendency of incidence and mortality for cervical cancer in Western Europe, possibly due to a well-established screening program, which allows for early diagnosis and treatment of cervical dysplasia/malignancy, the trend of the mortality rates for other HPV-associated diseases differs, even between countries with similar socioeconomic characteristics. The decreasing mortality rates for anal cancer reported in Germany may be due to the implementation of screening programs for high-risk populations [29], which may be non-existent or less well-established in other countries. A similar screening strategy has not been established for penile or female genitourinary cancer [30,31], which may account to a degree for the perceived differences. On the other hand, the incidence and outcome of HPV-related diseases depend on multiple factors to varying degrees, some of which are well-documented and others less so. These include but are not limited to the following preventive or permissive factors. Over the previous years, studies have reported a more permissive sexual behavior, increased smoking in older women cohorts, the aging population and an increased number of immunosuppressed individuals, new diagnostic tools and treatments [11,32,33], which may have had an impact on cancer rates and outcome to a different degree in different cohorts.

The average hospital stay for HPV-related cases decreases over time from 9 to 7 days, aligned harmoniously with the overall trajectory of average hospital stay, irrespective of admission cause. The decrease for the former was steady from 2000 to 2014, entered a stable phase from 2014 to 2019, followed by a steep decrease during the COVID-19 pandemic, from 2019 to 2021. This trend could be observed in all evaluated HPV-related diseases. Interestingly, the average hospital stay for female genitourinary tract cancer was the highest (9–15 days), twice as high as that for cervical cancer/dysplasia (5–8 days). The decline in average hospital stay can be attributed to a large degree to the implementation of the diagnostic-related group (DRG) system [34], but may also be partly due to an evolution of surgical techniques and treatment protocols.

Focusing on invasive and non-invasive cervical cancer, we observed a reduction in the hospitalization rates of both conditions. This reduction was observable in almost all age groups and in all German federal states, albeit at a varied degree. The increase in participation in screening programs in the 1990s and the implementation of vaccination programs since 2007 have probably contributed to the significant reduction in hospital admission rates [35]. The estimated coverage of cervical cancer screening in Germany was 81–92% [8]. A worldwide analysis has shown a declining cervical cancer risk in countries, where effective screening has been in place for a long time [36]. The vaccination coverage for HPV in Germany is 47% in the female population and only 5% in the male population [8], with a recent study reporting a significant lack of awareness of HPV and HPV vaccination [37]. Since the beginning of the vaccine implementation was not so long ago, the effects of the vaccine on the disease burden have not been fully evaluated until now. Interestingly, in our cohort, the 20–29 age group, which contains individuals who have been vaccinated against HPV, presented with a steep decreasing trend in the admission rates for cervical cancer. This could be an indication of the effectiveness of the vaccination program.

While the paper provides valuable insights into the burden of HPV-related hospitalization in Germany, it is important to acknowledge its limitations. Our analysis is based on health insurance data and thus includes only individuals who sought medical advice and were diagnosed with the relevant pathologies. Furthermore, the HPV attributable rate in different pathologies is calculated according to bibliographic estimates and not a direct examination of biological materials. The overall burden of HPV-related disease is surely underestimated, since our analysis does not include ambulatory care data, which are not available. Due to the limited resolution (three-character-ICD-10-GM) of the available data, our analysis could not include diagnoses such as genital warts, although HPV plays a central role in their pathology. That being said, the rarity of genital warts as a primary diagnosis in hospitalized patients makes their exclusion insignificant for our analysis (see Section 2). HPV has been associated with esophagus, larynx and skin cancer, but the association is more tenuous and less quantifiable compared to the well-established connection of HPV with the evaluated conditions [1], so these conditions could not be included in the calculation. Also coding errors in the insurance data cannot be excluded, but are expected to be minimal for such common diagnosis. The strengths of the study are the inclusion of the whole German population over a significant amount of time and that the study focuses on an important public health issue. This helps us avoid a possible sample selection bias and allows us to have a clearer picture of the evolution of HPV-related burden, in conjunction with the implemented screening and vaccination strategies.

This study has important implications for public health policymakers and healthcare providers in Germany. The decline in hospitalization rates for HPV-related diseases, particularly cervical cancer and dysplasia, suggests the potential efficacy of HPV vaccination and screening programs. The authors’ exploration of the impact of the COVID-19 pandemic on hospitalization rates highlights the need for adaptable healthcare strategies during unforeseen events.

In conclusion, our study provides a comprehensive analysis of the trends in HPV-related hospitalizations over the past two decades. The findings shed light on the changing landscape of HPV-related diseases in Germany and offer insights into the effectiveness of preventive measures such as screening and vaccination. This study contributes to the understanding of the burden of HPV-related diseases and can be of value to future public health initiatives in Germany and beyond.

## Figures and Tables

**Table 1:** Average annual percent change (AAPC) for invasive cervical cancer and carcinoma-in situ/cervical dysplasia from 2000–2001 for different age groups.

Age Group	AAPC	Confidence Interval
Invasive cervical cancer		
All women	**−1.89**	−2.07–−1.65
0–19 years	2.48	−22.62–35.56
20–29 years	**−4.86**	−5.83–−4.09
30–39 years	**−2.42**	−2.98–−1.52
40–49 years	**−2.78**	−3.14–−2.17
50–59 years	**−1.35**	−1.84–−0.85
60–79 years	**−1.62**	−1.86–−1.36
Over 80 years	**−2.25**	−2.84–−1.3
Carcinoma in situ/Dysplasia		
All women	**−4.91**	−5.34–−4.49
0–19 years	**−17.06**	−18.63–−15.45
20–29 years	**−12.17**	−12.91–−11.57
30–39 years	**−7.24**	−7.88–−6.51
40–49 years	**−3.43**	−4.05–−2.9
50–59 years	**−1.23**	−1.82–−0.65
60–79 years	**−1.64**	−2.32–−1.15
Over 80 years	0.21	−2–1.7

The bold marking indicates that the AAPC is significantly different from zero at the alpha = 0.05 level.

## Data Availability

Raw aggregated data are publicly available.

## References

[1] IARC (2012). IARC Monographs on the Evaluation of Carcinogenic Risks to Humans, Volume 100 B. A Review of Human Carcinogens: Biological Agents.

[2] RKI, Human Papillomviren. https://www.ecdc.europa.eu/en/human-papillomavirus.

[3] WHO, Human Papillomavirus (HPV). https://www.who.int/news-room/fact-sheets/detail/human-papilloma-virus-and-cancer.

[4] Plummer M., de Martel C., Vignat J., Ferlay J., Bray F., Franceschi S. (2016). Global burden of cancers attributable to infections in 2012: A synthetic analysis. Lancet Glob. Health.

[5] de Martel C., Plummer M., Vignat J., Franceschi S. (2017). Worldwide burden of cancer attributable to HPV by site, country and HPV type. Int. J. Cancer.

[6] Bray F., Ferlay J., Soerjomataram I., Siegel R.L., Torre L.A., Jemal A. (2018). Global cancer statistics 2018: GLOBOCAN estimates of incidence and mortality worldwide for 36 cancers in 185 countries. CA. Cancer J. Clin..

[7] Deleré Y., Remschmidt C., Leuschner J., Schuster M., Fesenfeld M., Schneider A., Wichmann O., Kaufmann A.M. (2014). Human Papillomavirus prevalence and probable first effects of vaccination in 20 to 25 year-old women in Germany: A population-based cross-sectional study via home-based self-sampling. BMC. Infect. Dis..

[8] Bruni L., Albero G., Serrano B., Mena M., Collado J.J., Gómez D., Muñoz J., Bosch F.X., Sanjosé S. ICO/IARC Information Centre on HPV and Cancer. Human Papillomavirus and Related Diseases in Germany. Summary Report 10 March 2023. https://hpvcentre.net/statistics/reports/DEU.pdf.

[9] Deleré Y., Meyer C., Reiter S. (2007). Universal immunisation with human papillomavirus (HPV) vaccine among females aged 12–17 recommended in Germany. Euro. Surveill..

[10] Reuschenbach M., Mihm S., Wölle R., Schneider K.M., Jacob C., Braun S., Greiner W., Hampl M. (2020). Burden of HPV related anogenital diseases in young women in Germany—An analysis of German statutory health insurance claims data from 2012 to 2017. BMC. Infect. Dis..

[11] Statistisches Bundesamt Deutschland. https://www-genesis.destatis.de/genesis/online.

[12] Giuliano A.R., Nyitray A.G., Kreimer A.R., Pierce Campbell C.M., Goodman M.T., Sudenga S.L., Monsonego J., Franceschi S. (2015). EUROGIN 2014 roadmap: Differences in human papillomavirus infection natural history, transmission and human papillomavirus-related cancer incidence by gender and anatomic site of infection. Int. J. Cancer.

[13] Kreimer A.R., Clifford G.M., Boyle P., Franceschi S. (2005). Human Papillomavirus Types in Head and Neck Squamous Cell Carcinomas Worldwide: A Systematic Review. Cancer Epidemiology. Biomark. Prev..

[14] Kuhdari P., Previato S., Giordani M., Biavati P., Ferretti S., Gabutti G. (2017). The burden of HPV-related diseases in Italy, 2001–2012. J. Public Health.

[15] Kim H.J., Fay M.P., Feuer E.J., Midthune D.N. (2000). Permutation tests for joinpoint regression with applications to cancer rates. Stat. Med..

[16] Joinpoint Regression Program, Version 5.0.2—May 2023; Statistical Methodology and Applications Branch, Surveillance Research Program, National Cancer Institute. https://surveillance.cancer.gov/joinpoint/.

[17] Restivo V., Minutolo G., Maranto M., Maiorana A., Vitale F., Casuccio A., Amodio E. (2023). Impact of Preventive Strategies on HPV-Related Diseases: Ten-Year Data from the Italian Hospital Admission Registry. Cancers.

[18] Restivo V., Costantino C., Amato L., Candiloro S., Casuccio A., Maranto M., Marrella A., Palmeri S., Pizzo S., Vitale F. (2020). Evaluation of the Burden of HPV-Related Hospitalizations as a Useful Tool to Increase Awareness: 2007–2017 Data from the Sicilian Hospital Discharge Records. Vaccines.

[19] Baldo V., Cocchio S., Buja A., Baldovin T., Furlan P., Bertoncello C., Saia M. (2013). Hospitalization for diseases attributable to human papillomavirus in the Veneto Region (North-East Italy). BMC Infect. Dis..

[20] López N., Gil-de-Miguel Á., Pascual-García R., Gil-Prieto R. (2018). Reduction in the burden of hospital admissions due to cervical disease from 2003-2014 in Spain. Hum. Vaccin. Immunother..

[21] Gil-Prieto R., Ester P.V., Álvaro-Meca A., Rodríguez M.S., De Miguel Á.G. (2012). The burden of hospitalizations for anus and penis neoplasm in Spain (1997–2008). Hum. Vaccines Immunother..

[22] Di Martino G., Cedrone F., Di Giovanni P., Tognaccini L., Trebbi E., Romano F., Staniscia T. (2023). The Burden of HPV-Related Hospitalizations: Analysis of Hospital Discharge Records from the Years 2015–2021 from a Southern Italian Region. Pathogens.

[23] Fu L., Tian T., Yao K., Chen X.F., Luo G., Gao Y., Lin Y.F., Wang B., Sun Y., Zheng W. (2022). Global Pattern and Trends in Penile Cancer Incidence: Population-Based Study. JMIR Public Health Surveill..

[24] Yang M., Du J., Lu H., Xiang F., Mei H., Xiao H. (2022). Global trends and age-specific incidence and mortality of cervical cancer from 1990 to 2019: An international comparative study based on the Global Burden of Disease. BMJ Open.

[25] Deshmukh A.A., Suk R., Shiels M.S., Sonawane K., Nyitray A.G., Liu Y., Gaisa M.M., Palefsky J.M., Sigel K. (2020). Recent Trends in Squamous Cell Carcinoma of the Anus Incidence and Mortality in the United States, 2001-2015. J. Natl. Cancer Inst..

[26] Damgacioglu H., Sonawane K., Zhu Y., Li R., Balasubramanian B.A., Lairson D.R., Giuliano A.R., Deshmukh A.A. (2022). Oropharyngeal Cancer Incidence and Mortality Trends in All 50 States in the US, 2001-2017. JAMA Otolaryngol. Head Neck Surg..

[27] Deng X., Liu Y., Zhan X., Chen T., Jiang M., Jiang X., Chen L., Fu B. (2022). Trends in Incidence, Mortality, and Survival of Penile Cancer in the United States: A Population-Based Study. Front. Oncol..

[28] Piechocki M., Koziołek W., Sroka D., Matrejek A., Miziołek P., Saiuk N., Sledzik M., Jaworska A., Bereza K., Pluta E. (2022). Trends in Incidence and Mortality of Gynecological and Breast Cancers in Poland (1980–2018). Clin. Epidemiol..

[29] S3-Leitlinie Analkarzinom Diagnostik, Therapie und Nachsorge von Analkanal und Analrandkarzinomen. Langversion 1.2-Dezember 2020. AWMF-Registernummer: 081/004OL. https://register.awmf.org/assets/guidelines/081-004OLl_Analkarzinom_Diagnostik-Therapie-Nachsorge-Analkanalkarzinom-Analrandkarzinom_2020-12.pdf.

[30] S3-Leitlinie Diagnostik Therapie und Nachsorge des Peniskarzinoms. Version 1.0-August 2020. AWMF-Registernummer: 043-042OL. https://register.awmf.org/assets/guidelines/043-042OLl_S3_Peniskarzinom_2020-08.pdf.

[31] Fink D., Ghisu G.P. Das Screening auf Zervix-, Vulva- und Analkarzinom. Schweizer Zeitschrift für Gynäkologie, 06.03.2020. https://www.rosenfluh.ch/media/gynaekologie/2020/01/Das-Screening-auf-Zervix-Vulva-und-Analkarzinom.pdf.

[32] Buttmann-Schweiger N., Klug S.J., Luyten A., Holleczek B., Heitz F., du Bois A., Kraywinkel K. (2015). Incidence patterns and temporal trends of invasive nonmelanotic vulvar tumors in Germany 1999–2011. A population-based cancer registry analysis. PLoS ONE.

[33] Wilking H., Thamm M., Stark K., Aebischer T., Seeber F. (2016). Prevalence, incidence estimations and risk factors of Toxoplasma gondii infection in Germany: A representative, cross-sectional, serological study. Sci. Rep..

[34] Reinhold T., Thierfelder K., Müller-Riemenschneider F., Willich S.N. (2009). Health economic effects after DRG-implementation—A systematic overview. Gesundheitswesen.

[35] Horn J., Damm O., Kretzschmar M.E., Deleré Y., Wichmann O., Kaufmann A.M., Garbe E., Krämer A., Greiner W., Mikolajczyk R.T. (2013). Estimating the long-term effects of HPV vaccination in Germany. Vaccine.

[36] Vaccarella S., Lortet-Tieulent J., Plummer M., Franceschi S., Bray F. (2013). Worldwide trends in cervical cancer incidence: Impact of screening against changes in disease risk factors. Eur. J. Cancer.

[37] Sharma S.J., Schartinger V.H., Wuerdemann N., Langer C., Möllenhoff K., Collin L., Sutton L., Riedl D., Kreuter A., Lechner M. (2022). Awareness of Human Papillomavirus (HPV) and HPV Vaccination amongst the General Population in Germany: Lack of Awareness and Need for Action. Oncol. Res. Treat..

